# Buck technique supplemented by temporary intersegmental pedicle screw fixation to repair lumbar spondylolysis in youth

**DOI:** 10.1186/s13018-024-04823-8

**Published:** 2024-06-08

**Authors:** Yuchen Ye, Huiwen Yang, Tao Ma, Kun Zhu, Gang Xu, Zhongbing Han, Zhili Zhang, Nan Wu, Xuan Guo, Huanyu Li, Pinghui Zhou, Zhengqi Bao, Changchun Zhang

**Affiliations:** 1 Department of Orthopaedics, The First Affiliated Hospital of Bengbu Medical University, 287 Changhuai Road, Bengbu, 233004 China; 2 Anhui Province Key Laboratory of Tissue Transplantation, Bengbu Medical University, 2600 Donghai Road, Bengbu, 233030 China; 3 The First Affiliated Hospital of Bengbu Medical University, 287 Changhuai Road, Bengbu, 233004 China

**Keywords:** Lumbar spondylolysis, Surgical repair, Buck technique, Intersegmental fixation, Sagittal balance, Herbert screw

## Abstract

**Background:**

Lumbar spondylolysis is a bone defect in the pars interarticularis of the lumbar vertebral, which is a common cause of low back pain in youth. Although non-surgical treatment is a mainstream option, surgery is necessary for patients with persistent symptoms. Buck technique is widely used as a classical direct repair technique, but it cannot achieve reduction of low-grade spondylolisthesis and reconstruction of lumbosacral sagittal balance. We have described a novel surgical procedure based on Buck technique with temporary intersegmental pedicle screw fixation, and report a series of clinical outcomes in 5 patients to provide a reference for the clinical treatment of young lumbar spondylolysis.

**Methods:**

Five young patients with symptomatic lumbar spondylolysis with a mean age of 19.20 ± 5.41 years underwent surgical treatment after an average of 7.60 ± 1.52 months of failure to respond to conservative treatment, using a new surgical procedure based on Buck technique combined with temporary intersegmental pedicle screw fixation.

**Results:**

Five patients were successfully operated without serious complications such as nerve and vascular injury. The average operation time was 109.00 ± 7.42 min, the interpretative average blood loss was 148.00 ± 31.14 ml, and the average fusion time was 11.20 ± 1.64 months. All patients were followed up for 2 years after surgery, and the visual analogue score (VAS) of low back pain and Oswestry disability index (ODI) scores were significantly improved compared with those before surgery, and the Henderson’s evaluation were rated excellent or good. After the removal of the internal fixation, it was observed that temporary intersegmental fixation could repair the isthmus, reduce lumbar spondylolisthesis, and reconstruct the sagittal balance of the lumbosacral vertebrae while preserving lumbar motion and preventing intervertebral disc degeneration. Postoperative MRI indicated the Pfirrmann classification of the affected discs: 1 case from grade III to grade II, 3 cases from grade II to grade I, and 1 case remained grade II.

**Conclusions:**

Buck technique supplemented by temporary intersegmental pedicle screw fixation is a highly applicable and effective method for the treatment of adolescent lumbar spondylolysis. The isthmic fusion is accurate, and temporary intersegmental fixation can effectively prevent disc degeneration and reconstruct the sagittal balance of lumbosacral vertebra.

## Introduction

**Fig. 1:**
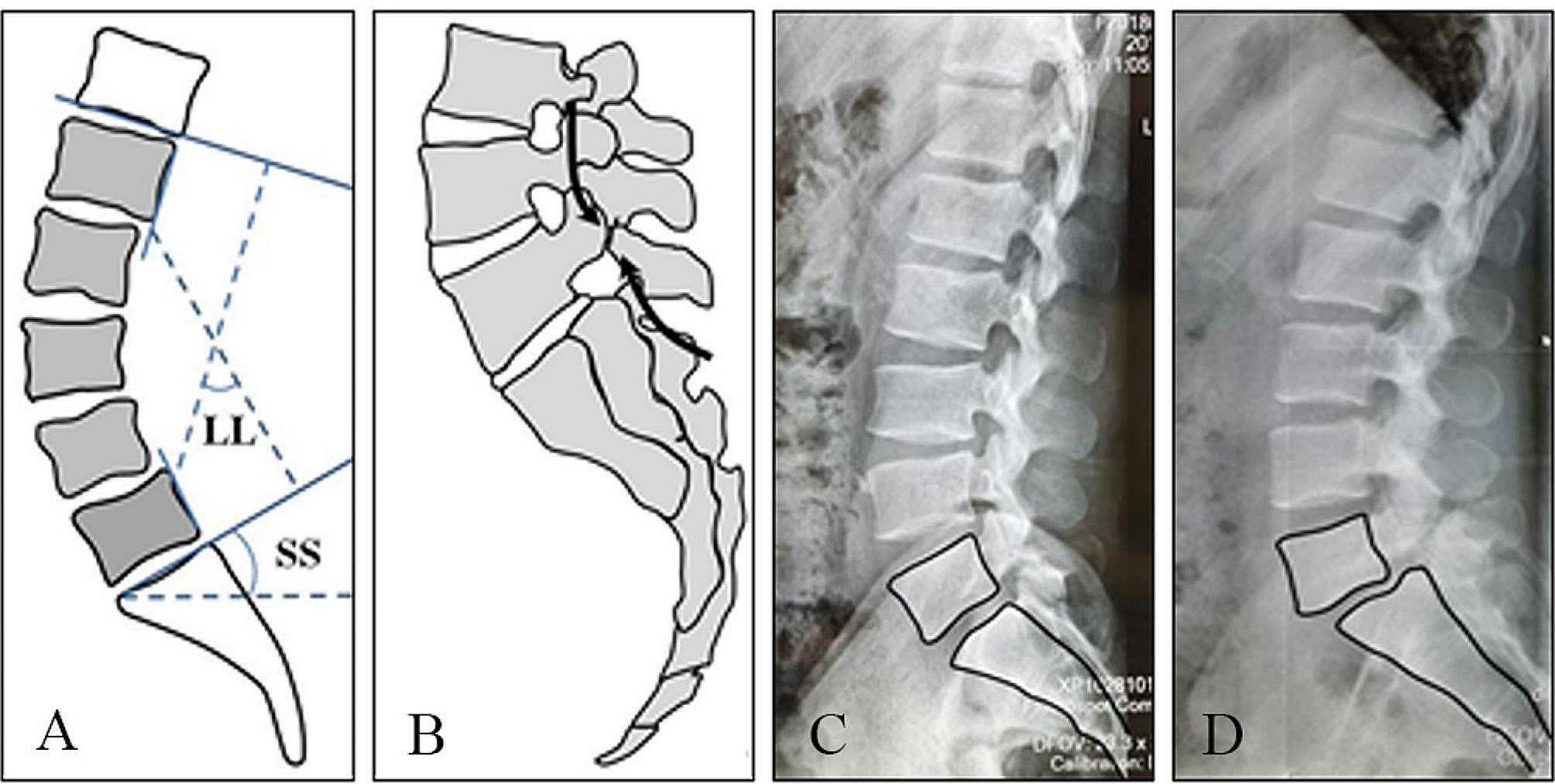
Schematic diagram of pathological mechanism and disease evolution of lumbar spondylolysis. (**A**) Lumbar lordosis and sacral slope. (**B**) The Lumbosacral stress is concentrated in the L5 pars interarticularis. (**C**, **D**) Lumbar spondylolysis eventually evolves into isthmus spondylolisthesis

Lumbar spondylolysis is a bone defect in the pars interarticulars (or isthmus) of the vertebral body. 95% of patients involve the interarticular (or isthmus) of the L5 and usually bilateral [1, 2]. The pathogenesis of lumbar spondylolysis is still controversial, but the most probable mechanism is that stress fractures can occur under the presence of high intensity and high frequency of lumbar activity in the congenital weak or dysplastic anatomic weak area of the vertebral spondylolysis [3, 4]. In addition, sagittal imbalances of the lumbosacral vertebrae (such as lumbar lordosis (LL) and sacral slope (SS)) can lead to stress concentration in the pars interarticularis, resulting in greater shear force on the isthmus and gradually develop into stress microfractures [5–8]. As stress continues, microfractures can make progress towards complete fractures and chronic nonunion of bone, and eventually to isthmus spondylolisthesis (Fig. 1). Therefore, youthful patients with symptomatic lumbar spondylolysis need surgical intervention if conservative treatment fails after a certain period of time.

At present, youthful patients with lumbar spondylolysis are often treated with isthmic repair, which is similar to conventional osteosynthesis [4, 9]. The purpose of the surgery is to relieve pain, stabilize the affected segment, promote the bony fusion of the pars interarticularis, rebuild the lumbosacral sagittal balance, and effectively prevent the occurrence of isthmic spondylolisthesis [10, 11]. According to previous reports, common isthmus repair methods mainly include Buck screw technique, Morscher hook screw technique, Scott steel wire technique, segmental pedicle screw lamina hook fixation technique etc [10, 12–14]. Buck technique has been widely applied because of its direct pressure on the isthmus and higher fusion rate. However, the Buck technique failed to address the stress concentration caused by lumbosacral sagittal imbalance, which resulted in an increased risk of complications related to internal fixation loosening, fracture, and poor bone fusion after isthmus repair. Therefore, we believe that intersegmental internal fixation is needed to adjust the LL and SS to reconstruct the sagittal balance of the lumbosacral vertebra and avoid the continuous concentration of stress in the pars interarticularis [15, 16]. 

**Fig. 2:**
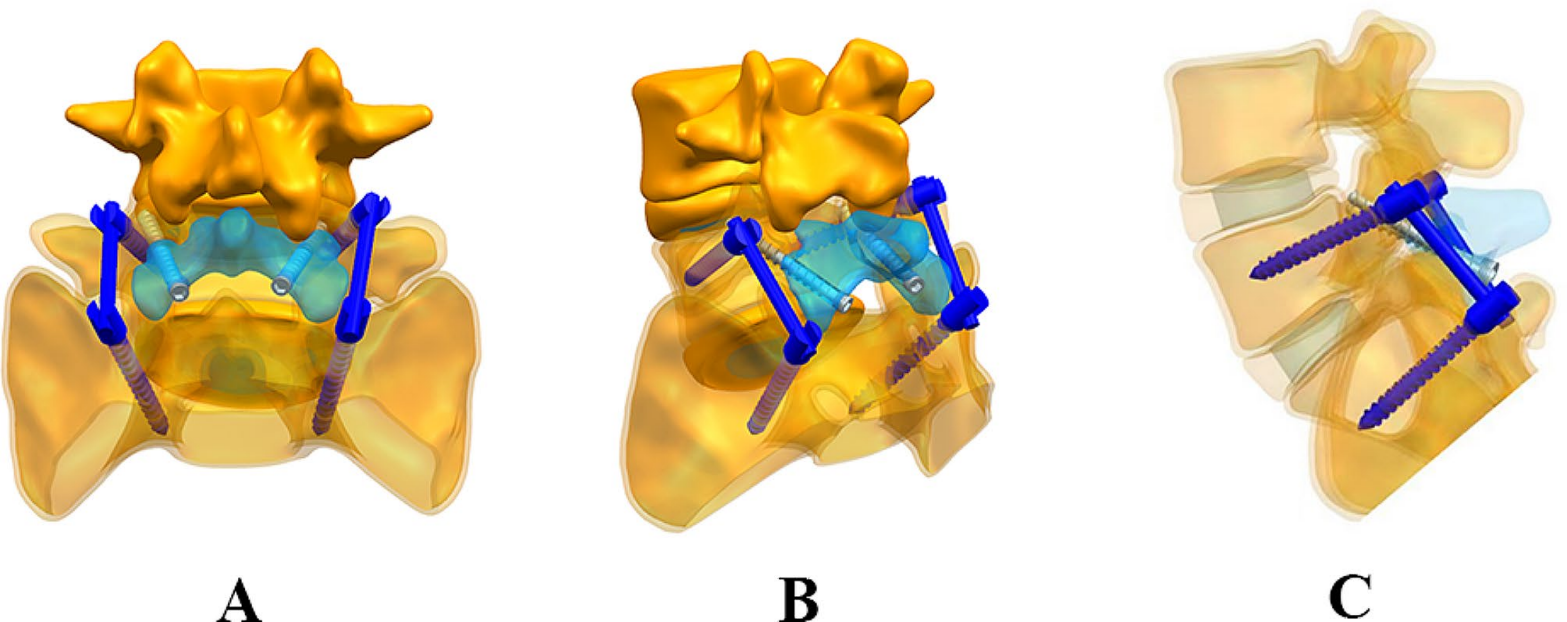
3D simulation of surgical procedure. (**A**) Main view. (**B**) Squint view. (**C**) Side view

In this study, we proposed a new surgical method for the treatment of lumbar spondylolysis based on Buck technique combined with temporary intersegmental pedicle screw fixation (Fig. 2). Based on the retrospective analysis of 5 patients, we evaluated the clinical effect of this method in the treatment of young lumbar spondylolysis.

## Methods

### Case presentation

**Fig. 3:**
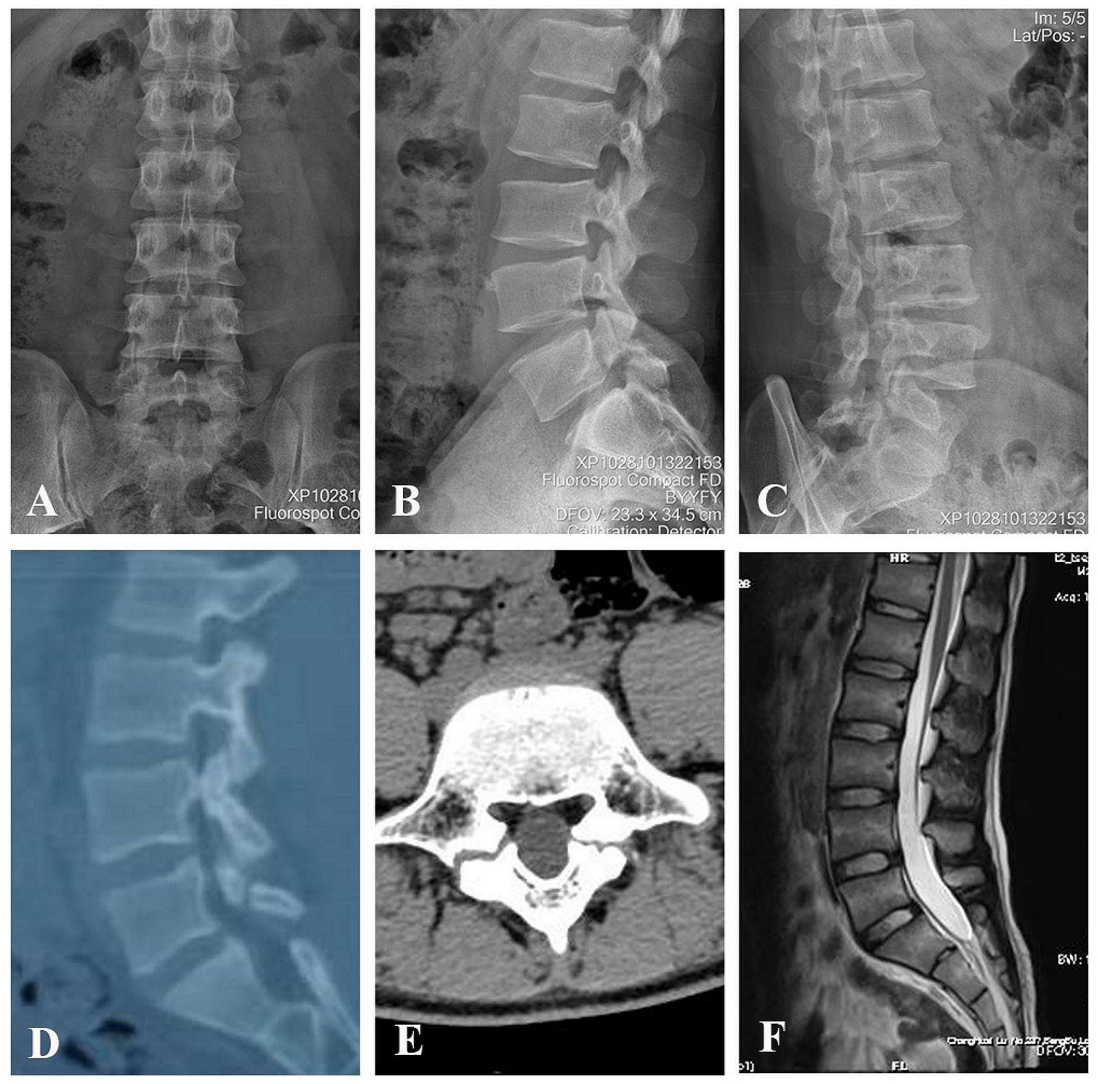
Case data Presentation. (**A**, **B**, **C**) X-ray images. (**D**, **E**) Computed tomography (CT) images. (**F**) Magnetic resonance imaging (MRI) pictures

Five patients were ranged from 10 to 25 years of age and presented with intractable low back pain that interfered with daily life. All patients initially underwent functional lumbar X-rays and underwent computed tomography (CT) and magnetic resonance imaging (MRI) to verify the diagnosis of lumbar spondylolysis (Fig. 3). At the same time, all patients received at least 6 months of conservative treatment with no significant relief of symptoms, requiring surgery due to serious impact on daily life. From 2019 to 2023, the five patients were successively operated by the same surgeon of the same institution and his operation team with Buck technique supplemented by temporary segmental pedicle screw fixation, and all operations were successfully completed. All the above procedures were approved by the bioethics Committee of the Medical College and the informed consent of the patient.

### Preoperative planning

According to the preoperative lumbosacral lateral radiographs or CT sagittal images, the degree of spondylolisthesis was evaluated and relevant sagittal parameters (such as LL and SS) were measured to make a personalized surgical plan, and the sagittal angle of pedicle screw placement and the pre-bending radian of the connecting rod were planned.

At the same time, the transverse diameter of the isthmus of the diseased vertebrae was measured according to the CT cross-sectional image, the direction of screw placement in the isthmus was designed with reference to the shape direction of the normal isthmus and the L5 / S1 intervertebral distraction angle, and the required amount of bone graft was estimated.

### Surgical procedure

#### Exposure and nail the pedicle screws

All patients were placed in the prone position after endotracheal intubation under aerostatic anesthesia. A posterior midline approach was implemented, dissecting and exposing the defective isthmus, lamina, bilateral facet joints and transverse processes along both sides of the spinous process. Special attention should be paid to the protection of supraspinal and interspinous ligaments and bilateral joint capsules. After intraoperative fluoroscopic positioning, pedicle screws’ nailing was performed in the isthmus defect vertebral body and the lower vertebral body. In order to minimize the stimulation and disturbance of the facet joint caused by the screw tail, the screw placement points were slightly laterally compared with the Weinstein method.

#### Repair of isthmus and iliac bone transplantation

All scar tissue and hyperplastic osteophytes around the isthmus defect around the broken end of the isthmus were completely removed, at the same time fully loosen the isthmus end and retain normal structure. Confirm whether spondylolisthesis is reduced and sacral slope corrected by intraoperative fluoroscopy. Under the same skin incision, the corresponding volume of bone mass was taken within the posterior superior iliac spine according to the range of the isthmus bone defect at this time. After clipped and compacted, iliac bone was implanted in the gap bilaterally.

#### Buck screw placement

The transition of the lower margin of the lamina and the lower margin of the spinous process was selected as the entry point, and the direction of the screw placement was from the middle of the inner and outer cortex of the lamina towards the superior articular process of the affected vertebra. A guidance needle (φ = 1 mm) was inserted along the longitudinal axis of the pars interarticularis, extending upward and forward about 40° according to the direction of the isthmus of the upper vertebral body. After the position was satisfied, two Herbert screws (φ = 3 mm) was inserted, and the two sides were alternately tightened and compressed to restore the isthmus length and width.

#### Intersegmental fixation

**Fig. 4:**
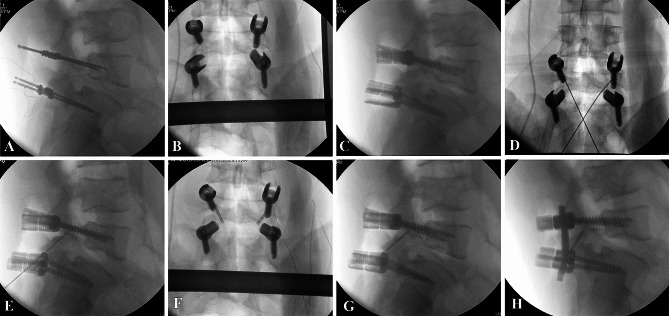
Details of the surgical procedures. (**A**, **B**, **C**) Exposure and nail the pedicle screws. (**D**, **E**, **F**) Repair of isthmus, iliac bone transplantation and nail the Herbert srews. (**G**, **H**) Intersegmental fixation and reconstruct sagittal balance

After pre-bending and installing the connecting rod, the vertebral body was reduced by intersegmental fixation and the lumbosacral sagittal balance was reconstructed. Finally, it was confirmed by fluoroscopy that the isthmus bone graft was sufficient, the length and width were restored, the spondylolisthesis was basically reduced, and the SS and LL angles were corrected satisfactorily. The last fluoroscopy confirmed that the isthmus bone graft was enough, the spondylolisthesis was basically reduced, and the sagittal balance was reconstructed satisfactorily (Fig. 4).

### Postoperative treatment and follow-up

Within 24 h after operation, routine intravenous injection of non-steroidal anti-inflammatory drugs for analgesia and first-generation cephalosporin for anti-infection treatment. Both drugs were applied according to the instructions. Pay close attention to the patient’s pain condition and incision status within 48 h after operation, and add analgesics and strengthen incision nursing if necessary. On the 3rd postoperative day, the patient could walked out of bed with a lumbar brace and underwent anteroposterial-lateral lumbar radiographs to re-evaluate the isthmus repair and internal fixation position.

After 3 days, patients can be discharged according to their own conditions and then treated at home under the guidance of rehabilitation therapists or transferred to rehabilitation department for further treatment. Patients can come out of bed under the protection of lumbar brace for limited time functional exercise, maintain a relatively resting state, avoid doing physical labor for 3–4 weeks.

In convalescence, the patient should go to the outpatient clinic for monthly review. The patient should be evaluated by the surgeon and the rehabilitation physician for many times and be guided by the rehabilitation training. CT scans were performed at 4 and 6 months to assess isthmus fusion and fixation status. Then, make a rehabilitation plan according to the specific situation and gradually resume daily activities. Moreover, preoperative and postoperative disc conditions were evaluated using the Pfirrmann classification [17]. 

### Postoperative evaluation

The surgical outcomes were evaluated, including operation time, blood loss and fusion time. For radiological evaluation, the bone fusion of isthmus defect was evaluated by ordinary X-ray and CT, and the degree of disc degeneration was evaluated by MRI. VAS score, ODI score and Henderson’s evaluation of functional capacity were utilized to compare the symptoms before and after operation [18]. 

## Results

All 5 patients were presented with bilateral spondylolysis at L5 level, and their conservative treatment lasted an average of 7.60 ± 1.52 months without satisfactory results. The mean intraoperative blood loss was 148.00 ± 31.14 ml, and the mean fusion time was 11.20 ± 1.64 months. The fusion standard of bone transplantation was that continuous bone morphology or bone trabecular appeared in the fusion area of imaging plane, and the isthmus fracture line disappeared. All 5 patients were followed up for 2 years. (Table 1)

**Table 1:** Characteristics of patients including postoperative outcomes

Case	Age (Years)/Sex	Pars Lysis Level	Laterality	Conservative Treatment Duration (Months)	OP time(min)	Blood loss(ml)	Fusion time (months)
1	17/M	L5	Bilateral	6	110	120	13
2	20/F	L5	Bilateral	9	100	130	9
3	24/M	L5	Bilateral	8	105	150	10
4	25/M	L5	Bilateral	6	120	200	12
5	10/F	L5	Bilateral	9	110	140	12
Mean ± SD	19.20±5.41			7.60 ± 1.52	109.00 ± 7.42	148.00 ± 31.14	11.20 ± 1.64

The VAS of low back pain and the ODI scores improved significantly compared with those before internal fixation. The internal fixation device was removed and a radiographic overstretch test was performed to observe the level of activity during the fixation phase. Preoperative MRI examination showed that 1 case was grade III and 4 cases were grade II. Postoperative MRI indicated the Pfirrmann classification of the affected discs: 1 case from grade III to grade II, 3 cases from grade II to grade I, and 1 case remained grade II. In terms of Henderson’s evaluation, three patients were rated excellent and the remaining two was rated good. (Table 2)

**Table 2:** Evaluation of surgical procedure

Case	VAS					ODI			Pfirrmann classification			Henderson
	Pre-OP		Last follow-up			Pre-OP	Last follow-up		Pre-OP	Last follow-up		
1	7		1			35	6		II	I		Excellent
2	8		1			33	5		III	II		Excellent
3	7		2			32	9		II	I		Good
4	7		1			33	8		II	II		Excellent
5	7		2			34	5		II	I		Good
Mean ± SD	7.20 ± 0.55		1.40 ± 0.55			33.40 ± 1.14	6.60 ± 1.82					

## Discussion

Lumbar spondylolysis is a widespread disease and the main cause of low back pain in young people. It most commonly is present at the L5 segment bilaterally [19]. Currently, there are two main types of repair operations for spondylolysis: one is direct repair using segmental internal fixation and bone grafting in the isthmus; the other is fusion of the affected vertebra with adjacent vertebrae using intersegmental internal fixation. For patients with simple spondylolysis without obvious spondylolysis, various surgical methods such as Buck screw technique and pedicle screw-hook technique are often used. However, these internal fixation methods are non-intersegmental and therefore cannot solve problems such as lumbar spondylolysis and lumbar-sacral sagittal imbalance. For lumbar spondylolysis with spondylolisthesis in youth, the previous experience was to perform interbody fusion by removing the disc. However, interbody fusion may sacrifice a motor unit to induce adjacent segment degeneration, which is often an awful outcome for young people. Faced with possible complications, many scholars have modified isthmus repair surgery. For example, Huang et al. proposed a surgical method of isthmic bone graft repair combined with temporary intersegmental pedicle screw [15], and Berjano et al. proposed a novel technique with pedicle screws, rod and polyester band [20]. However, the author believes that all the above surgical methods have advantages and disadvantages.

Based on the underlying pathological mechanism of lumbar spondylolysis and previous literature reports, the author believes that the key to the repair of lumbar spondylolysis lies in: (1) The hyperplasia tissue at the broken end of the isthmus should be fully removed and bone graft be performed according to the scope of the bone defect. The direct fixation of the isthmus was realized by using compression screw technology, so as to restore the continuity and integrity of the bone while ensuring the stability of the isthmus [12, 13]; (2) For mild lumbar spondylolisthesis and lumbosacral sagittal imbalance, we need to use intersegmental fixation to reduce spondylolisthesis and rebuild the spine-pelvis balance, so as to avoid the stress concentration on the intervertebral disc or isthmus area which resulting in disc degeneration and poor bone fusion [15, 16]. 

**Fig. 5:**
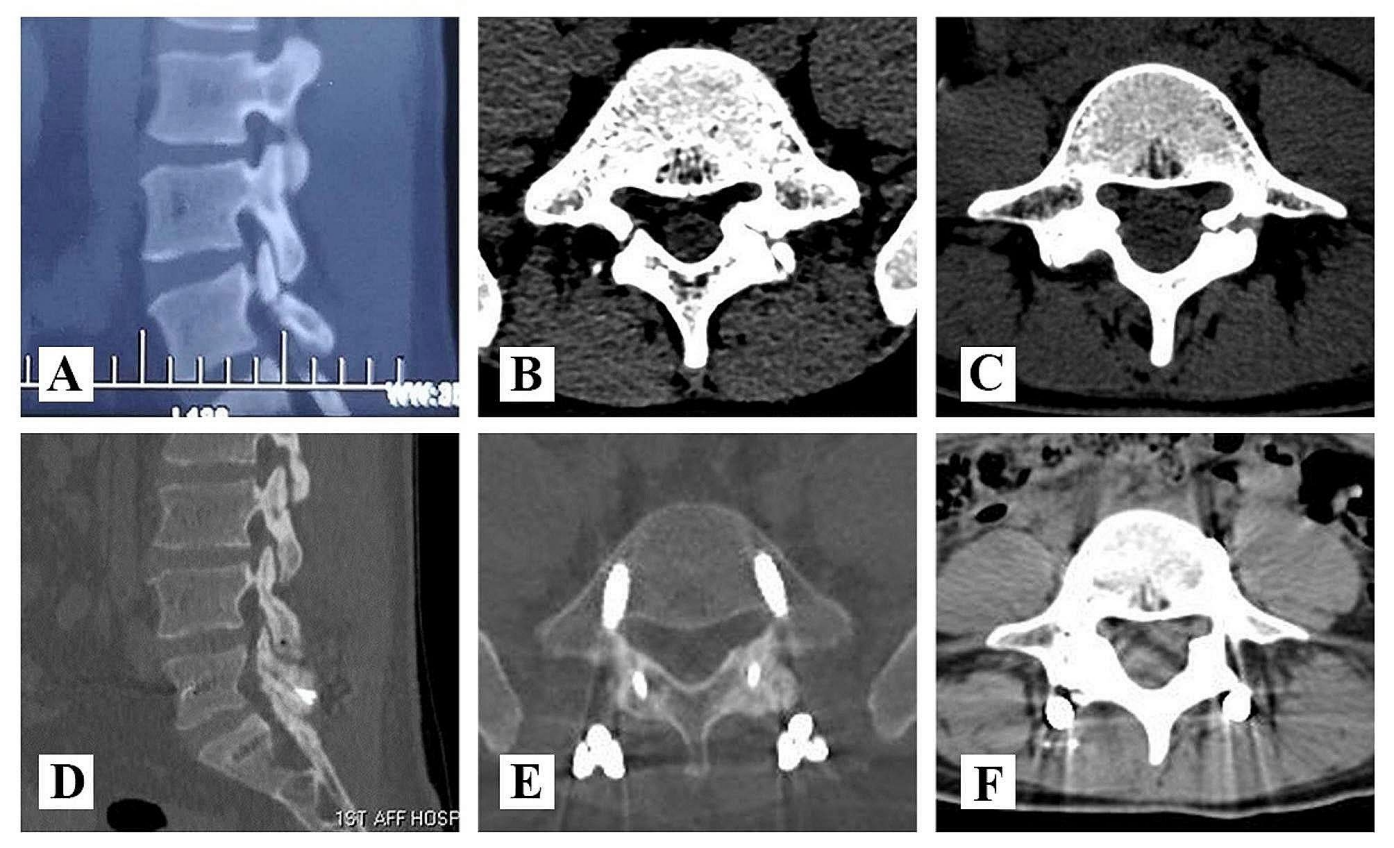
CT indicated satisfactory isthmus fusion. (**A**, **B**, **C**) Preoperative CT imaging data of the patient. (**D**, **E**, **F**) Postoperative CT showed bony fusion

**Fig. 6:**
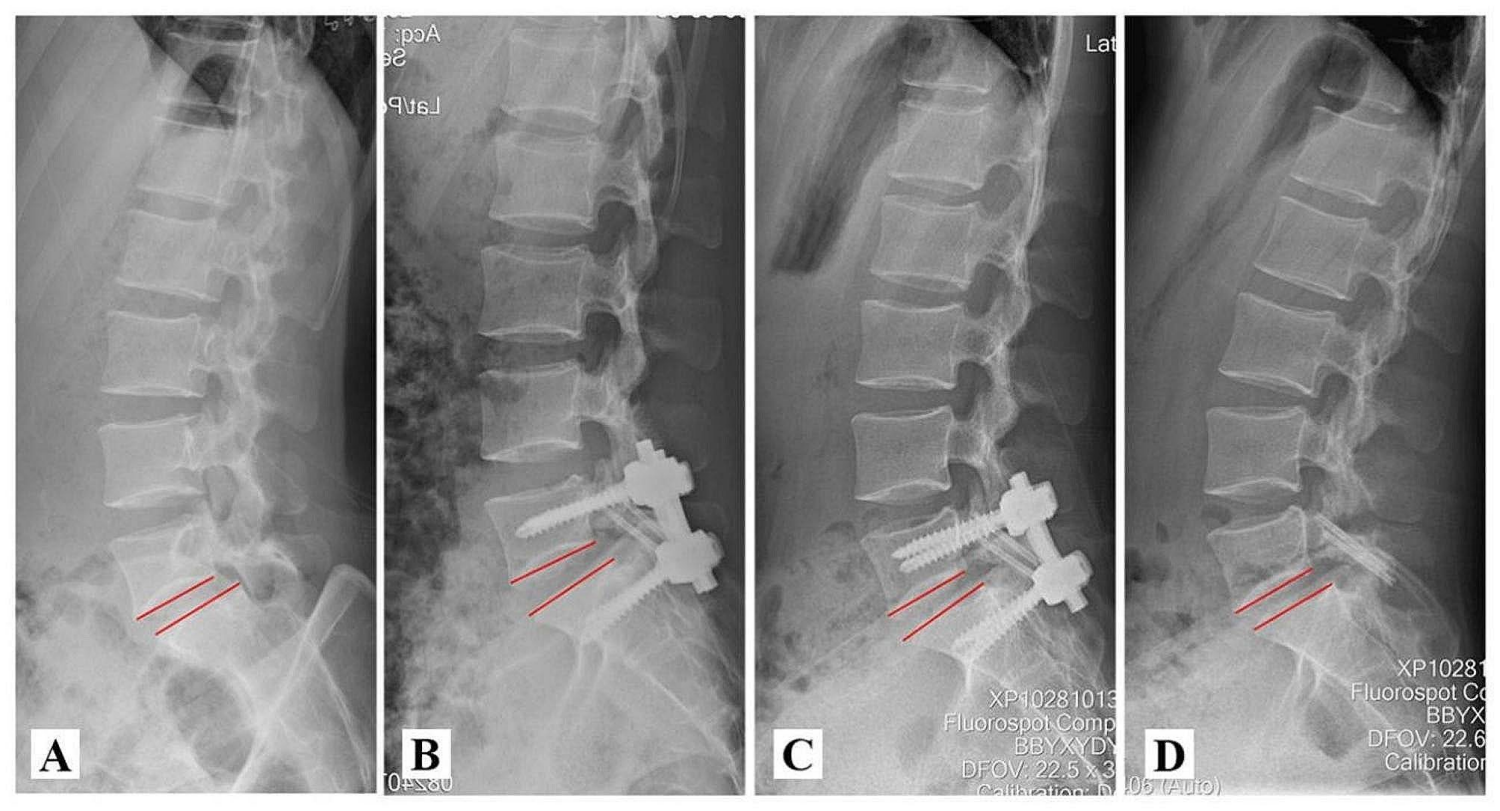
X-ray follow-up showed that intersegmental fixation could restore lumbosacral sagittal balance. (**A**) Preoperative. (**B**) Postoperative Day 3. (**C**) 6 months after surgery. (**D**) One year after surgery, the internal fixation was removed

**Fig. 7:**
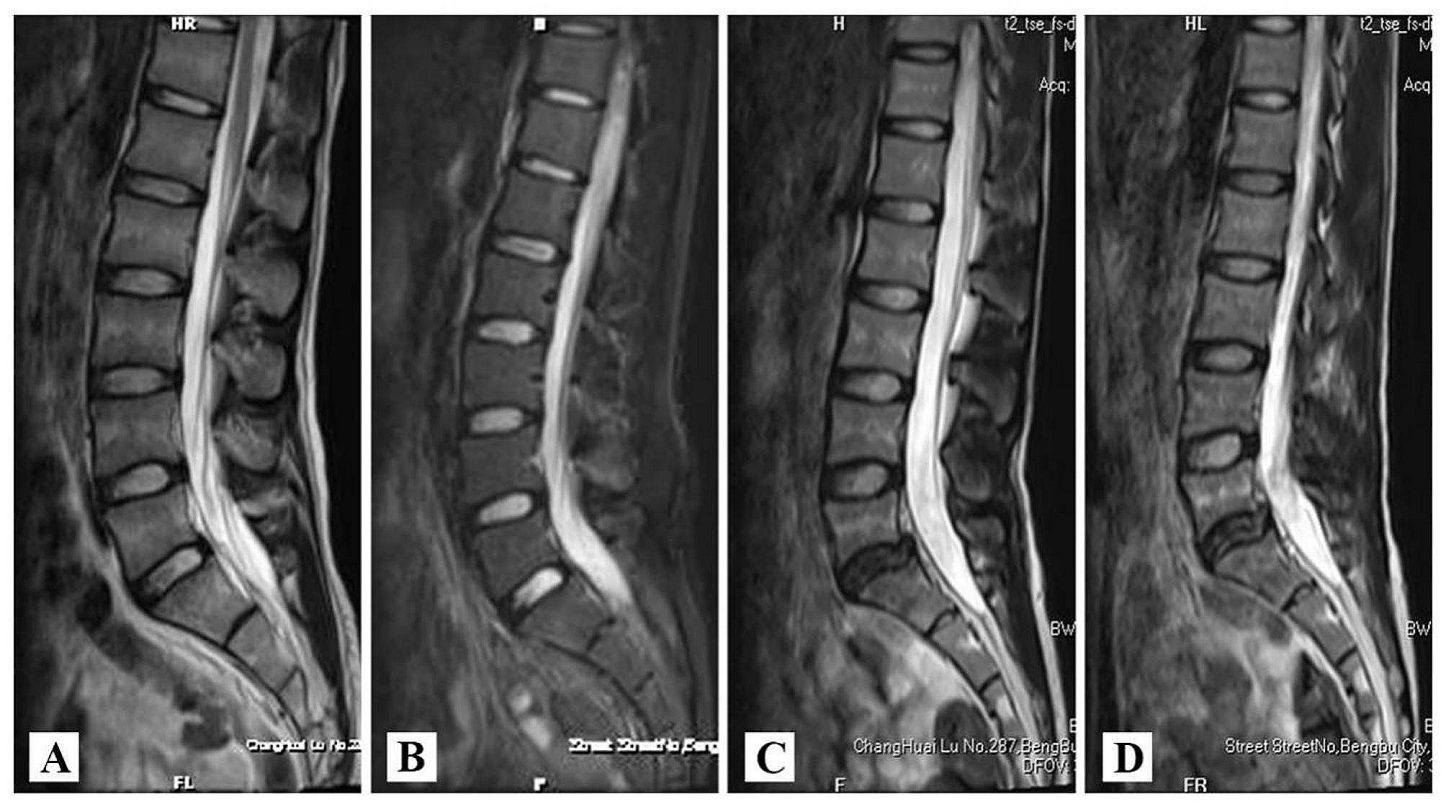
MRI indicated the Pfirrmann classification of the affected discs: (**A**, **B**) One case from grade II to grade I; (**C**, **D**) Another case from grade III to grade II

According to the above ideas, we proposed a novel technique for spondylolysis repair based on Buck technique supplemented by temporary segmental pedicle screw fixation. By comparing the CT images before and after the operation, we can confirm that Buck technique can directly repair the isthmus defect with bone grafting and restore the complete lumbar posterior arch (Fig. 5). Meanwhile, we can observe based on radiographs that temporary intersegmental fixation played a significant role on the reconstruction of sagittal balance of the lumbosacral vertebra (Fig. 6). It can reduce stress concentration on the defect area of the pars interarticularis and indirectly promote bone fusion. In particular, considering that the Buck technique directly occupied a portion of the bone graft space through the isthmus section, we modified the details of the procedure to reduce the occupying effect of the Buck screw by replacing a slightly smaller diameter Herbert screw(φ = 3 mm), which have many advantages for repairing nonunion or stress fractures [21, 22]. In addition, in order to prevent the degeneration of intervertebral disc or adjacent segments which caused by long-term intersegmental fixation, patients will be regularly followed up after surgery (Fig. 7). Within 1 year of healing of the lumbar isthmic fracture, pedicle screws will be removed to obtain better ROM values, during which time rehabilitation will be strengthened to restore as much range of motion in the lumbar spine as possible [23]. 

While Buck technique’s effectiveness is undisputed, the most controversial aspect of the technique involves the temporary fixation of moving segments with intersegmental pedicle screws. Here we have our opinions as follows. As a three-column spinal fixation, the biomechanical properties of pedicle screws should better control intersegmental extension and rotational stresses [24]. There was a very interesting clinical and biomechanical study showed that the spondylolysis originates ventrally in the interarticular region, simply because higher stresses were found in the ventral caudate during repeated hyperextension and rotation activities in all loading modes [25]. At the same time, some studies show that the imbalance of lumbosacral sagittal position may play an important role in the pathological process of lumbar spondylolysis in adolescent population [5, 6, 26]. LL and SS were positively correlated with lumbar spondylolisthesis rate in that an excessively large angle of LL and SS leads to lumbar center of gravity moves forward, while the support point of gravity moves backward [7]. The stress of lumbosacral will be concentrated in the isthmus of the 5th lumbar vertebra, resulting in a large shear force on the isthmus. Loss of sagittal balance in the lumbosacral region and morphological abnormalities may be responsible for high involvement of L5. High frequency and intensity of stress applied to the lumbar spondylolisthesis can lead to the gradual progression of the spondylolisthesis from microfractures to complete fractures and chronic nonunion. Moreover, Jeon et al. believed that pedicle screw fixation in a short period of time did not affect the recovery of lumbar motion [27]. Therefore, we can conclude that temporary intersegmental internal fixation is feasible for the repair of isthmus.

Absolutely, our current research also has many limitations and deficiencies. First of all, the number of case samples in this study is insufficient, so we need more patients and control groups in the later study to compare the therapeutic effect of the current proposed technology with that of the traditional technology. Second, this new surgical method requires two surgeries to complete, and the treatment cycle may be too long to meet the needs of some young patients for rapid recovery. In addition, the implantation of pedicle screws decreased the mobility of the adjacent segments of the segmental fixation. A biomechanical validation of this condition was done in our other article. In the short-term follow-up, we did not find significant degeneration of the disc in the proximal stage in five patients. However, we determined from our biomechanical analysis that there may be a potential risk of disc degeneration in the adjacent segment with this reduced mobility [28]. Finally, the Buck technique requires difficult nailing techniques and extensive surgical experience, so it is hoped that this kind of surgery can be carried out smoothly in the future with the assistance of computer navigation and neural monitoring technology [29]. 

## Conclusion

Buck technique supplemented by temporary intersegmental pedicle screw fixation is a highly applicable and effective method for the treatment of adolescent lumbar spondylolysis. The Buck technique can confirm a high healing rate of the pars interarticularis, and temporary intersegmental fixation can effectively prevent disc degeneration and reconstruct the sagittal balance of lumbosacral vertebra. Thus, we believe that this novel surgical technique is worthy of clinical application and promotion.

## Data Availability

No datasets were generated or analysed during the current study.
